# Real world risk of infusion reactions and effectiveness of front-line obinutuzumab plus chlorambucil compared with other frontline treatments for chronic lymphocytic leukemia

**DOI:** 10.1186/s12885-022-09256-2

**Published:** 2022-02-06

**Authors:** Nicole Bourrier, Ivan Landego, Oliver Bucher, Mandy Squires, Erin Streu, Irena Hibbert, Theresa Whiteside, Spencer B. Gibson, Marc Geirnaert, James B. Johnston, David E. Dawe, Versha Banerji

**Affiliations:** 1 Max Rady College of Medicine, 727 McDermot Ave, Winnipeg, MB R3E 3P5 Canada; 2 Department of Internal Medicine, Rady Faculty of Health Sciences, University of Manitoba, 727 McDermot Ave, Winnipeg, MB R3E 3P5 Canada; 3 Department of Epidemiology, CancerCare Manitoba, 675 McDermot Ave, Winnipeg, MB R3E 0V9 Canada; 4 CancerCare Manitoba Research Institute, 675 McDermot Ave, Winnipeg, MB R3E 0V9 Canada; 5 Department of Nursing, CancerCare Manitoba, 675 McDermot Ave, Winnipeg, MB R3E 0V9 Canada; 6 Department of Biochemistry and Medical Genetics, Max Rady College of Medicine University of Manitoba, 727 McDermot Ave, Winnipeg, MB R3E 3P5 Canada; 7 Department of Immunology, Max Rady College of Medicine University of Manitoba, 727 McDermot Ave, Winnipeg, MB R3E 3P5 Canada; 8 Provincial Oncology Drug Program, CancerCare Manitoba, 675 McDermot Ave, Winnipeg, MB R3E 0V9 Canada; 9 Department of Medical Oncology and Hematology, CancerCare Manitoba, 675 McDermot Ave, Winnipeg, MB R3E 0V9 Canada

**Keywords:** Chronic lymphocytic leukemia, CLL, Obinutuzumab, Front-line therapy, Retrospective cohort study

## Abstract

**Background:**

Chronic lymphocytic leukemia (CLL) is the most common type of leukemia in North America. Previous studies have shown improved progression free survival (PFS) and response rates in unfit patients treated with obinutuzumab compared to other regimens. The aim of this study was to evaluate the obinutuzumab-chlorambucil regimen in the context of historical treatments and first-dose infusion reactions at CancerCare Manitoba (CCMB).

**Methods:**

A retrospective chart review was conducted for patients treated with obinutuzumab from January 1, 2014 to December 31, 2017 at CCMB. A minimum data set was extracted for patients treated with other front-line therapies. Descriptive statistics were used to evaluate patient demographics, toxicity, duration and dosing of obinutuzumab treatment. Kaplan–Meier curves were used to evaluate time-to-next-treatment (TTNT), overall survival (OS) and PFS for patients treated with obinutuzumab. A multivariable logistic regression model was used to investigate associations between infusion related reactions (IRRs) and age at treatment, pre-treatment lymphocyte count, cumulative illness rating scale (CIRS) and receipt of prior chemotherapy.

**Results:**

Forty seven percent of patients receiving frontline therapy received chlorambucil and obinutuzumab. Sixty-seven patients were treated with obinutuzumab and consisted of 36 males (53.7%) and 31 females (46.3%) with 29 patients (43.3%) over age 75 years. Rates of grade 3 and 4 obinutuzumab IRRs were lower (6%) compared to the CLL11 clinical trial (20%) due to local practices including slower infusion rates and using chlorambucil before starting obinutuzumab treatment. Many patients had difficulty tolerating the full dosage of chlorambucil. Only 26 patients (38.8%) had their dose of chlorambucil escalated to the full dose of 0.5 mg/kg. In addition, only 18 patients (26.9%) received all doses of obinutuzumab and all 12 doses of chlorambucil.

**Conclusions:**

In summary, first dose infusion reactions with obinutuzumab can be markedly reduced by using chlorambucil to decrease the lymphocyte count before obinutuzumab and by using a very slow initial obinutuzumab infusion rate. Modifications in chlorambucil dosing and obinutuzumab administration can improve tolerance without significant loss in efficacy.

**Supplementary Information:**

The online version contains supplementary material available at 10.1186/s12885-022-09256-2.

## Introduction and background

### Chronic lymphocytic leukemia burden and treatment

Chronic lymphocytic leukemia (CLL) is the most common type of leukemia in North America with an estimated 21,040 new cases and 4,060 deaths in the United States in 2020 [1]. In Manitoba (population 1.36 million in 2020), over 100 new cases are diagnosed each year [2, 3]. CLL is more common in older adults, with a median age at diagnosis between 67 and 72 differing between clinic specific versus population-based studies [4, 5]. The burden of this disease is likely to increase with the current aging population [4].

CLL is characterized by an accumulation of malignant clonal B cells in the blood, lymph nodes, bone marrow and spleen [4]. Patients with asymptomatic disease are monitored and treatment is initiated when either the disease advances or the patient becomes symptomatic [4]. The standard therapy for CLL has been chemoimmunotherapy where patients with immunoglobulin heavy chain variable region gene (*IgHV*) mutated disease can achieve prolonged remissions, but most patients will have a clinical course punctuated by remissions and relapses [4]. Patients with advanced age and multiple comorbidities requiring CLL treatment have an increased risk of complications and poorer overall prognosis [6]. The choice of treatment is based on patient age, functional status, renal function, molecular markers and comorbidities [4].

The CIRS score is an important tool for measuring the number and severity of comorbidities [7]. For patients with CLL, CIRS scores have guided management. Patients who are considered fit as measured by a CIRS score of less than or equal to 6, an ECOG performance status of 0–2 and normal renal function are treated with more intensive regimens such as fludarabine-cyclophosphamide-rituximab (FCR), fludarabine-rituximab (FR) (< 65 years) or bendamustine-rituximab (BR) (> 65 years) [8]. Unfit patients historically received single agent chlorambucil [4]. Obinutuzumab is a humanized, glycoengineered antibody directed against CD20 [9]. This monoclonal antibody has enhanced antibody-dependent cellular toxicity and direct cell death compared to rituximab [9]. A large randomized phase 3 trial (CLL11) demonstrated that unfit patients (CIRS > 6 or estimated creatinine clearance of 30-69 ml per min) treated with obinutuzumab (OB) plus chlorambucil (CLB) experience longer progression free survival and higher rates of complete response compared to chlorambucil-rituximab (CLB/R) and chlorambucil alone. [9]

Obinutuzumab is known to cause infusion-related reactions (IRRs). As compared to rituximab, obinutuzumab is unique in directly causing cell death, by inducing lysosomal membrane permeabilization with cathepsin release and free radical formation [10]. This leads to a large release of cytokines from tumor cell lysis and therefore these reactions are associated with increased white blood cell count and/or tumor burden [11]. These reactions can be mild (grade 1–2) or severe (grade 3–4) and can even rarely lead to death (grade 5) [11]. To prevent IRRs from occurring, patients are premedicated with antihistamines and steroids prior to receiving obinutuzumab [11].

The aim of this study was to investigate the real-world experience of obinutuzumab with chlorambucil, by studying patients treated through the CLL Clinic at CCMB. CCMB is an academic centre that is ideal for population-based, real-world evidence studies because it is the referral centre for the entire province of Manitoba.

## Methods

### Patient selection

We completed a retrospective chart review of all CLL patients starting front-line therapy that included obinutuzumab in the Canadian province of Manitoba from January 1, 2014 to December 31, 2017, identified through the CLL CAISIS database which captures patients from our centralized CLL outpatient clinic. Molecular stratification including fluorescence in situ hybridization (FISH) became available in Manitoba during 2016. Some patients with high risk disease (del11q or del17p) still received obinutuzumab and chlorambucil based on patient or physician preference and were included in our analysis. Patient demographics, patterns of treatment, reported toxicities and grading, response rates and survival data were collected. IRR’s were graded using the National Cancer Institute Common Terminology Criteria for Adverse Events version 5.0 and neutropenia, anemia and thrombocytopenia were graded using the scale provided in the iwCLL guidelines [12, 13]. A minimum data set was extracted from the CAISIS database for patients starting other front-line therapies (CLB/R, CLB, BR, fludarabine-rituximab (FR) and FCR) during the above time period.

## Treatment

Obinutuzumab in combination with chlorambucil has been funded by the Manitoba healthcare system for first-line treatment of CLL since June 22, 2015 [14]. For cycle 1, obinutuzumab was given intravenously at a dose of 100 mg on day 1, 900 mg on day 2, and 1000 mg on days 8 and 15 [15]. For cycles 2–6, obinutuzumab was given at a dose of 1000 mg on day 1 [15]. Each of these were considered a dose. At CCMB, patients are started at an initial rate of 6 mg/hr on day 1 and this rate is doubled every hour to a max rate of 24 mg/hr [15]. The duration for the infusion is 325 min for day 1, 240 min for day 2 and 195 min for each subsequent dose [15]. Chlorambucil was administered at a reduced dose (0.25 mg/kg) on days 1 and 15 of cycle 1 compared to the CLL11 clinical trial [9]. Chlorambucil was given at a dose of 0.5 mg/kg on days 1 and 15 of cycles 2–6 if the lower dose was well tolerated [15]. Therefore, patients ideally receive a total of 8 doses of obinutuzumab and 12 doses of chlorambucil. However, if patients experienced significant toxicity, chlorambucil doses were held or reduced. Patients who received single agent obinutuzumab (*n* = 11) were included with the obinutuzumab-chlorambucil patients for the analyses.

In this study, hematologic response was defined when hemoglobin > 100 g/L, platelets > 100 × 10^9^/L, lymphocytes < 5 × 10^9^/L and neutrophils > 1 × 10^9^/L as most patients did not have imaging studies after completing treatment. Progression was defined as a lymphocyte count greater than 5 × 10^9^/L, progression on CT scan or signs of relapse on physical examination such as lymphadenopathy or splenomegaly.

## Statistical analysis

Descriptive statistics were used to compare demographics of individuals treated with obinutuzumab to those treated with other first-line regimens (CLB/R, CLB, BR, FR, and FCR) and to evaluate the toxicity, duration and dosing of obinutuzumab treatment. We compared tolerability of obinutuzumab therapy based on age (< 75 and ≥ 75) and CIRS score (< 8 and ≥ 8) using Chi-square tests, with *P*-values ≤ 0.05 indicative of statistical significance. We chose 8 as the cutoff for high and low CIRS scores as it was the median CIRS for the obinutuzumab group.

Kaplan–Meier curves, stratified by treatment regimen, were used to evaluate TTNT for each of the regimens. TTNT was defined as the time from the start of first-line therapy to the start of second-line therapy or death whichever occurred first. Censoring occurred at data cut-off (July 5, 2019). Differences between chlorambucil containing regimens were tested using a log-rank test. *P*-values ≤ 0.05 were considered statistically significant. The same methods, stratified by age and CIRS, were used to evaluate TTNT and OS for the obinutuzumab cohort. Differences between age groups and CIRS score categories were tested using a log-rank test with *P*-values ≤ 0.05 considered statistically significant. OS was defined as the time from start of treatment with obinutuzumab to death. A PFS analysis by Kaplan Meier for patients treated with obinutuzumab was conducted. The date of progression was the date of clinical or hematologic progression (as defined above) or death.

A multivariable logistic regression model was used to investigate associations between IRRs and age at treatment, pre-treatment lymphocyte count, CIRS score and receipt of prior chemotherapy. Univariable associations between each variable of interest and IRRs were tested using likelihood ratio testing to screen variables for inclusion into the multivariable model. Variables with *P*-values ≤ 0.2 were eligible for inclusion in the multivariable model. These variables were also screened for potential multicollinearity using Spearman’s rank correlation and Point-Biserial correlation coefficients. Coefficients with values ≥ 0.80 were considered indicative of multicollinearity. Sensitivity analyses were used to assess confounding between all variables of interest with changes to regression coefficients ≥ 20% indicative of confounding. Potential interactions between each of the variables of interest were added to the final model if coefficients from the interaction term were statistically significant (P-values were ≤ 0.05). The multivariable model was built using likelihood ratio testing with *P*-values ≤ 0.05 indicative of significance. Continuous variables were modeled using restricted cubic splines, with Akaike’s information criteria used to determine the number of knots included in the spline function. Pearson and deviance residuals as well as leverage and delta-beta values were generated to check for influential observations. The Hosmer–Lemeshow test was used to test model Goodness-of-fit.

## Results

### Patient demographics and clinical characteristics

We reviewed 142 patients starting front-line therapies (OB/CLB, CLB/R, CLB, BR, FR, and FCR) between January 1, 2014 – December 31, 2017 and found 47% were treated with obinutuzumab (Table 1). As expected, since obinutuzumab is used in unfit patients, those receiving this regimen were older (median age at treatment = 73, range = 55–98) and had higher CIRS scores (median CIRS = 8, range = 3–15) than those treated with fludarabine-based regimens (median age FR = 70, median CIRS FR = 6, median age FCR = 62, median CIRS FCR = 3). We did not observe large variations in the prognostic markers for each treatment group.

**Table 1:** Patient and disease characteristics at the time of treatment for CLL patients by front-line regimen

Characteristic		OB/CLB	CLB/R	CLB	BR	FR	FCR
		**(*n* = 67)**	**(*n* = 14)**	**(*n* = 11)**	**(*n* = 5)**	**(*n* = 8)**	**(*n* = 37)**
**Age**	**Median (range)**	73 (55–98)	82 (69–90)	79 (57–87)	76 (62–77)	70 (48–83)	62 (35–62)
	**75 + **	29 (43.3)	12 (85.7)	8 (72.7)	3 (60.0)	2 (25.0)	0 (0.0)
	**Under 75**	38 (56.7)	2 (14.3)	3 (27.3)	2 (40.0)	6 (75.0)	37 (100.0)
**Sex**	**Female**	31 (46.3)	5 (35.7)	5 (45.4)	3 (60.0)	5 (62.5)	13 (35.1)
	**Male**	36 (53.7)	9 (64.3)	6 (54.6)	2 (40.0)	3 (37.5)	24 (64.9)
**Stage**	**Rai 0**	2 (3.0)	0 (0.0)	3 (27.3)	0 (0.0)	0 (0.0)	1 (2.7)
	**Rai 1**	6 (9.0)	4 (28.6)	0 (0.0)	0 (0.0)	1 (12.5)	17 (46.0)
	**Rai 2**	12 (17.9)	3 (21.4)	2 (18.2)	1 (20.0)	3 (37.5)	10 (27.0)
	**Rai 3**	18 (26.9)	3 (21.4)	5 (45.4)	1 (20.0)	1 (12.5)	0 (0.0)
	**Rai 4**	17 (25.4)	2 (14.3)	1 (9.1)	1 (20.0)	1 (12.5)	3 (8.1)
	**SLL**	12 (17.9)	2 (14.3)	0 (0.0)	2 (40.0)	2 (25.0)	6 (16.2)
**CIRS score**	**Median (range)**	8 (3–15)	7 (2–15)	10 (3–14)	5 (3–7)	6 (1–10)	3 (0–10)
**CD38 status**^**a**^	**Positive**	32 (47.8)	5 (35.7)	5 (45.4)	4 (80.0)	3 (37.5)	19 (51.4)
	**Missing**	4 (6.0)	0 (0.0)	0 (0.00)	0 (0.0)	0 (0.0)	0 (0.0)
**Zap70 status**^**a**^	**Positive**	15 (22.4)	11 (78.6)	4 (36.4)	4 (80.0)	4 (50.0)	21 (56.8)
	**Missing**	15 (22.4)	0 (0.0)	0 (0.0)	0 (0.0)	0 (0.0)	0 (0.00)
**IgVH Mutation**	**Unmutated**	22 (32.8)	3 (21.4)	2 (18.2)	3 (60.0)	4 (50.0)	18 (48.7)
	**Missing**	25 (37.3)	6 (42.9)	5 (45.4)	1 (20.0)	1 (12.5)	9 (24.3)
**FISH Status**	**High Risk**^**b**^	8 (11.9)	0 (0.0)	3 (27.3)	1 (7.1)	7 (18.9)	1 (12.5)
	**Missing**	20 (29.9)	3 (60.0)	5 (45.4)	7 (50.0)	10 (27.0)	3 (37.5)
**ALC**	**Median (range)**	44.4 (0.5–336.1)	54.4 (1.1–258.4)	71.3 (14.2–599.5)	7.6 (1.5–419.9)	74.8 (1.0–192.3)	89.8 (1.0–429.8)
**HGB**	**Median (range)**	110 (73–169)	109 (83–149)	103 (81–137)	127 (82–129)	114 (90–138)	130 (60–162)
**Platelets**	**Median (range)**	134 (13–687)	132.5 (59–280)	132 (65–179)	164 (120–285)	116.5 (79–341)	143 (9–254)
**B2M**	**n**	48	9	8	4	3	13
	**Median (range)**	4.6 (2.0–14.4)	4.6 (2.8–16.7)	4.3 (2.8–14.1)	4.6 (3.0–6.3)	3.7 (2.5–3.8)	3.5 (2.4–5.5)

*OB/CLB* obinutuzumab-chlorambucil, *CLB/R* chlorambucil-rituximab, *CLB* chlorambucil, *BR* – bendamustine-rituximab, *FR* fludarabine-rituximab, *FCR* fludarabine-cyclophosphamide-rituximab, *IgHV* immunoglobulin heavy chain variable region gene, *ALC*  absolute lymphocyte count, *HGB*   hemoglobin, *B2M*  beta 2 microglobulin

^a^ value at diagnosis (positive defined as ≥ 20% positive)

^b^ high risk FISH: 11q23 deletion and 17p13 deletion

### Obinutuzumab patient characteristics

A total of 67 patients treated with obinutuzumab met inclusion criteria (Table 1). This cohort consisted of 36 males (53.7%) and 31 females (46.3%). In addition, 29 patients (43.3%) were aged 75 years or more. The median age at diagnosis was 68 (range: 46–94) and the median age at treatment was 73 (range: 55–98). The median CIRS score at the time of treatment was 8 (range: 3–15). The median CIRS score was 8 (range: 3–15) for the patients under 75 and 7 (range: 4–14) for those over 75.

We found lower lymphocyte counts prior to treatment in the obinutuzumab cohort compared to other regimens (Table 1). Of note, there were 16 patients in our cohort that received treatment with low dose chlorambucil prior to receiving treatment with obinutuzumab due to early experience of IRRs and a possible association with higher lymphocyte counts.

### Uptake of treatment

The number of patients being treated with this regimen increased from 9 patients in 2015 to 30 patients in 2016 to 26 patients in 2017.

### Safety

#### Infusion-related reactions

There were 29 patients (43.4%) that had an IRR on the first day of treatment (25 grade 2 reactions, 3 grade 3 reactions and 1 grade 4 reaction). The rate of reactions was numerically higher in the over 75 age group and in patients with a CIRS score below 8, but neither reached statistical significance (Table 2).

**Table 2:** Adverse reactions based on age and CIRS score

Toxicity	Total	Age at Treatment			CIRS		
		**75 + **	**Under 75**	***P*-value**^**a**^	**8 + **	**Under 8**	***P*-value**^**a**^
**Infusion-related reaction**	29 (43.28)	14 (48.28)	15 (39.47)	0.47	14 (37.84)	15 (50.00)	0.32
**Neutropenia**	41 (61.19)	18 (62.07)	23 (60.53)	0.90	24 (64.86)	17 (56.67)	0.49
**Anemia**	25 (37.31)	14 (48.28)	11 (28.95)	0.11	15 (40.54)	10 (33.33)	0.54
**Thrombocytopenia**	42 (62.69)	19 (65.52)	23 (60.53)	0.68	20 (54.05)	22 (73.33)	0.10
**Infections**	28 (41.79)	10 (34.48)	18 (47.37)	0.29	16 (43.24)	12 (40.00)	0.79

^a^ Chi-square *P*-value

There was a subset of 16 patients that received pre-treatment with 2 to 6 mg/day of chlorambucil from 6 days to 3 months duration to reduce the lymphocyte count prior to starting obinutuzumab. These patients had a lower mean lymphocyte count (34.17 × 10^9^, standard deviation (SD): 48.3) just prior to obinutuzumab administration and a marginally lower rate of IRRs (37.5%) compared to those with no prior chlorambucil (mean lymphocyte count: 65.81 × 10^9^, SD: 69.81, rate of IRRs: 42%). All the infusion reactions were grade 2 or less in the patients who received chlorambucil pre-treatment.

Using logistic regression, univariable associations between each variable of interest and IRRs were tested and only pre-treatment lymphocyte count was found to be statistically significant, *P*-value of 0.023 (Table 3). Further modeling showed that higher pre-treatment lymphocyte count increases the risk of an IRR, until the IRR plateaus as the lymphocyte count increases above 100 (Supplementary Figure 1).

**Table 3:** Multivariable model to predict infusion reactions

**Variable**		**Odds ratio**	**Standard error**	**95% confidence interval**		***P*-value**	**Overall *P*-value**
**Age at treatment**	-	0.999	0.029	0.945	1.057	0.977	0.977
**Lymphocyte count** **(pre-treatment)**	-	1.009	0.005	1.001	1.018	0.037	0.023
**CIRS score at treatment**	-	0.89	0.089	0.731	1.083	0.245	0.234
**Prior treatment**	Remote treatment	Reference					
	Prior treatment	0.73	0.43	0.231	2.313	0.593	0.591

#### Tolerability of treatment

While 38 patients (56.7%) received all 8 doses of obinutuzumab, only 18 patients (26.9%) received all doses of obinutuzumab and all 12 doses of chlorambucil. The median number of chlorambucil doses given was 10 (range: 1–12) and when stratified by age the median was 8 (range: 1–12) in those aged over 75 years and 10.5 (range: 1–12) for those aged under 75 years (Table 4). Only 26 patients (38.8%) had their dose of chlorambucil escalated to the full dose of 0.5 mg/kg (Table 4). Three patients were started at the full dose because they were enrolled in a clinical trial and the remaining 23 patients who achieved full dose had their dose increased during treatment (15 increased with cycle 2, 5 increased with cycle 3, 2 increased with cycle 4, and 1 increased with cycle 5). The remaining patients remained at a reduced dose (30 patients (44.8%)) or did not receive any chlorambucil (11 patients (16.4%)) throughout all six cycles of obinutuzumab treatment (Table 4). A higher proportion of patients over the age 75 (79.3%) did not achieve the full dose of chlorambucil compared to those under 75 (47.4%, *P*-value < 0.01) (Table 4). CIRS score did not appear to impact likelihood of patients achieving the full dose of chlorambucil (Table 4).

**Table 4:** Treatment with obinutuzumab-chlorambucil based on age and CIRS score

Treatment characteristic	Total	Age at treatment			CIRS		
		75 +	Under 75	*P*-value	8 +	Under 8	*P*-value
Achieved full dose of chlorambucil	26 (38.81)	6 (20.69)	20 (52.63)	< 0.01^a^	15 (40.54)	11 (36.67)	0.75^a^
Completed 6 cycles of obinutuzumab	46 (68.66)	18 (62.07)	28 (73.68)	0.31^a^	25 (67.57)	21 (70.00)	0.83^a^
Number of chlorambucil doses, median (range)	10 (1–12)	8 (1–12)	10.5 (1–12)	0.06^b^	10 (1–12)	9 (1–12)	0.78^b^
Number of obinutuzumab doses, median (range)	9 (1–9)	8 (1–9)	9 (4–9)	-	9 (2–9)	9 (1–9)	-

^a^ Chi-square *P*-value

^b^ Wilcoxon-Mann Whitney *P*-value

Treatment with obinutuzumab and chlorambucil was discontinued prior to completing all six cycles in 21 patients (31.3%) due to toxicity, patient or provider preference. Treatment completion was similar in both age and CIRS groups (Table 4). Considering the population is often older and higher comorbidity we assessed an age cut off of 85 or a CIRS score of 10. Fewer patients aged 85 years or more successfully received full dose chlorambucil or obintuzumab, however with the small sample size significance was not achieved (Supplementary Table 1).

### Treatment efficacy

#### Response, overall survival and progression free survival of the whole OB/CLB cohort

A total of 51 patients (76.1%) achieved a hematologic response. There were no statistically significant differences in response rate between ages 75 + or less than 75 years (72.41 v 78.95%, *p* = 0.53) and CIRS 8 + or less than 8 (70.27 v 83.33%, *p* = 0.21).

**Fig. 1:**
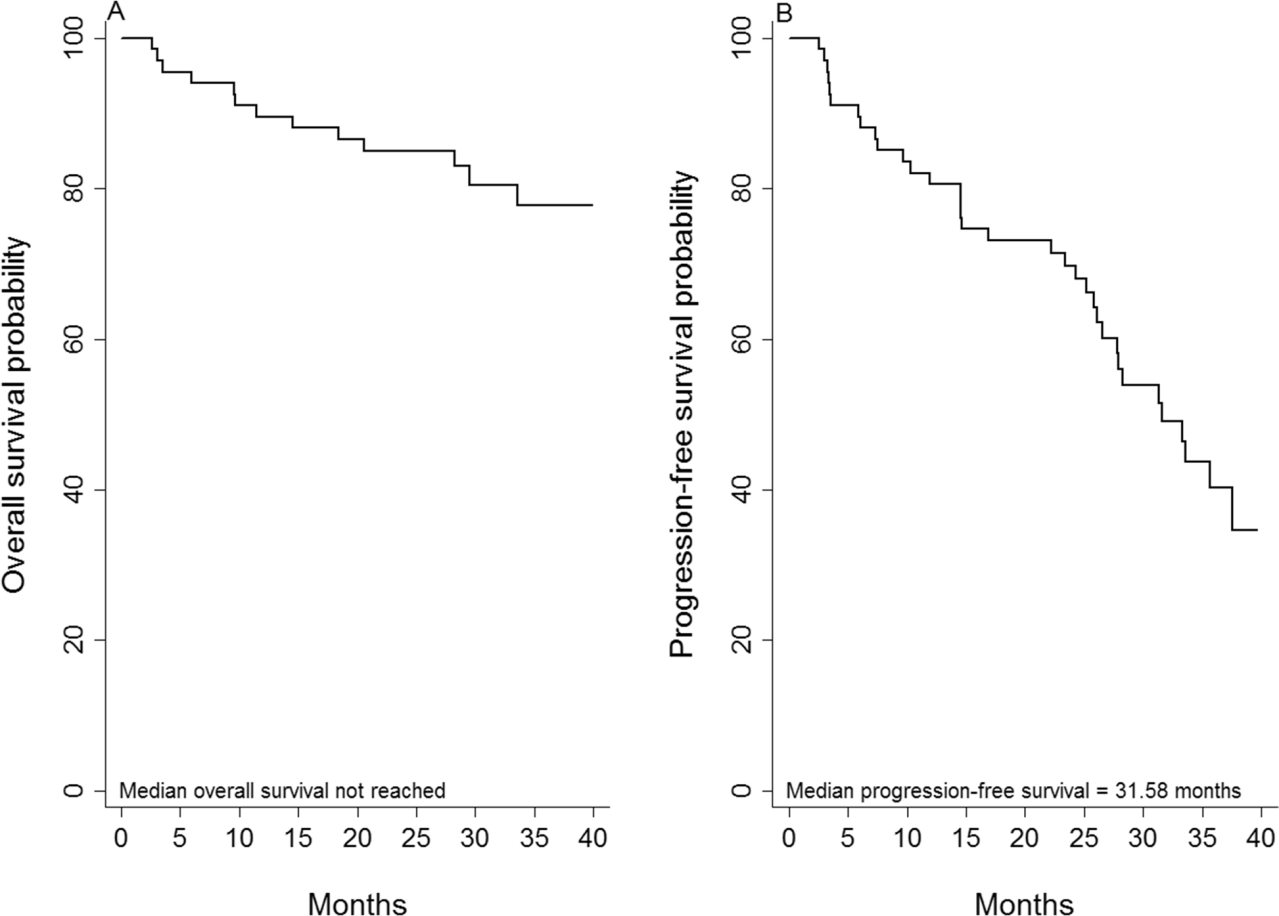
Overall survival (OS) and Progression free  survival (PFS) of the whole cohort to death

Median OS for the obinutuzumab cohort has not been reached. Overall survival at 35 months follow-up was approximately 80% (Fig. 1A). As shown in Fig. 1B, more than 60% of patients progressed at 40 months.

#### Time to next treatment

**Fig. 2:**
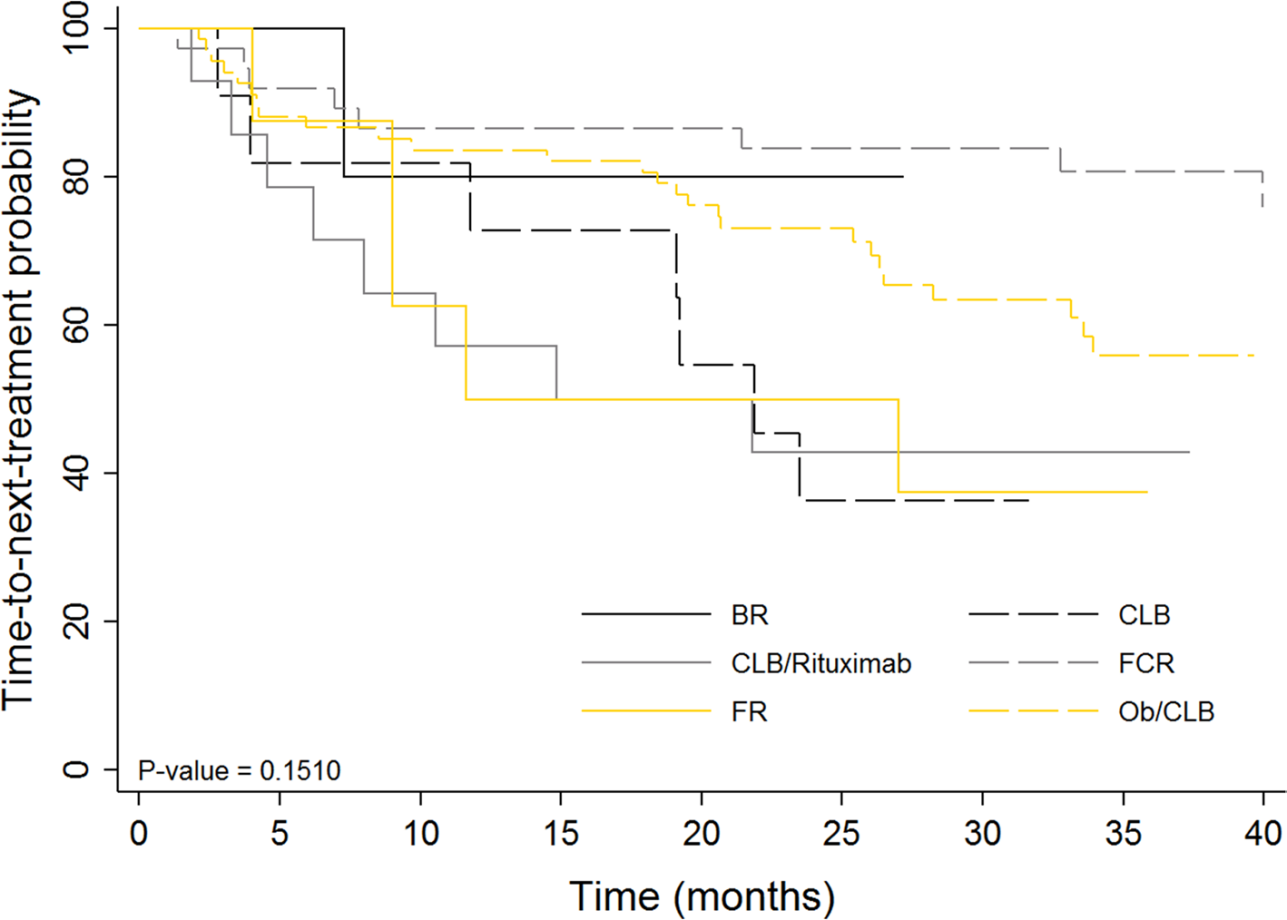
Time to next treatment (TTNT): Kaplan–Meier curves of time to next treatment, stratified by treatment regimen

**Fig. 3:**
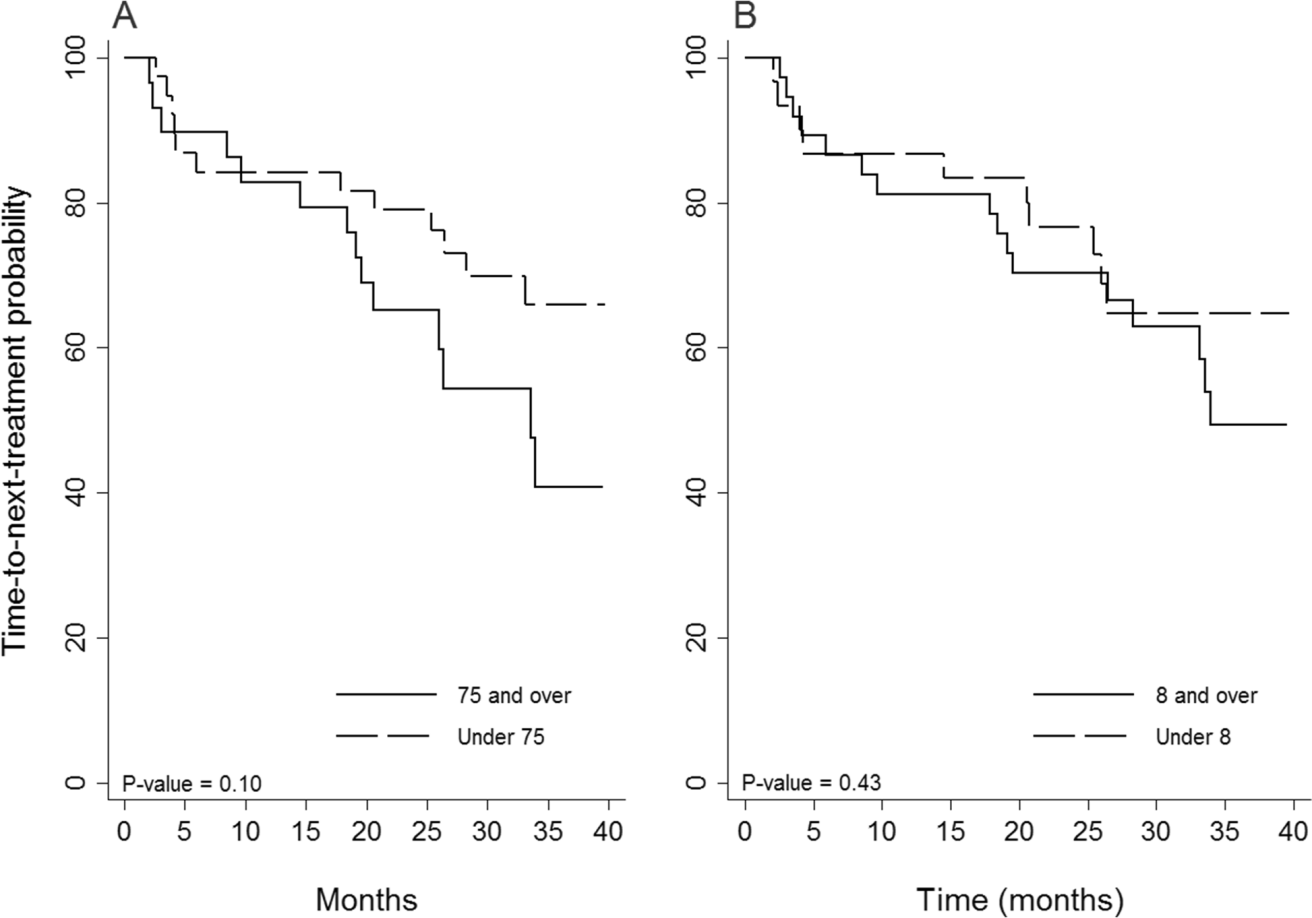
Kaplan–Meier curves of TTNT stratified by age (**A**) (*P*-value = 0.10) and CIRS score (**B**) (*P*-value = 0.43)

Figure 2 demonstrates the TTNT curve for the OB/CLB group compared to other regimens. The TTNT curve was also stratified by age (Fig. 3A) and CIRS score (Fig. 3B) for the OB/CLB cohort. A larger proportion of patients in the older age group have died or required additional treatment, but this difference was not statistically significant (*p *= 0.10). We did not see a difference based on CIRS score. The PFS is shorter than the TTNT, as there are several patients who demonstrated signs of relapse but have not met criteria for second-line treatment.

## Discussion

Compared to the CLL11 clinical trial, our obinutuzumab cohort was similar in both age and CIRS score (CLL11: median age (range) = 74 (39–89), median CIRS (range) = 8 (0–22)) [9]. A real world study of obinutuzumab-chlorambucil from the Polish Adult Leukemia Group also exhibited similar patient characteristics (median age = 74, median CIRS = 8) [16]. However, our CLB/R and CLB cohorts were much older (CLL11: CLB/R median age = 73, CLB median age = 72 versus Manitoba Cohort: CLB/R median age = 82, CLB median age = 79) and the CLB group had higher CIRS scores than the clinical trial (CLL11: CLB median CIRS = 8 versus Manitoba Cohort: CLB median CIRS = 10) [9].

Very few patients received BR in Manitoba during the time period for this study and these patients were found to be older compared to the literature (BR literature: median age = 72) [17]. Fludarabine regimens are reserved for patients who are young and fit. Our FCR cohort had similar age and CIRS compared to the literature (FCR: median age = 61, median CIRS = 1 versus Manitoba Cohort FCR: median age = 62, median CIRS = 3) [18]. Our FR cohort was older compared to the literature (FR: median age = 63 (CALGB 9712), 64 (CALGB 9011), 70 (Manitoba Cohort)) [19].

A higher proportion of patients treated with CLB, FR and CLB/R received additional treatment compared to the other regimens (Fig. 1). Contrary to expectations, FR and CLB/R did not exhibit longer TTNT compared to single agent chlorambucil, though group numbers were small. Approximately 36% of the patients that received FR and CLB/R did not complete the full treatment and this may have contributed to the poor response. In the obinutuzumab treatment group, the 75 + age group appeared to have shorter TTNT, but the comparison was not statistically significant. Patients in this group may be relapsing sooner due to less treatment with chlorambucil. Patients with more comorbidity did not require second-line treatment sooner than patients with low CIRS scores (Fig. 3).

Rates of grade 3 and 4 IRRs were lower in our cohort (6%) compared to the CLL11 study (20%) [9]. The original protocol of the CLL11 study was to administer 1000 mg for the first dose of obinutuzumab [20]. The infusion was started at an initial rate of 50 mg/hr and increased by 50 mg/hr every 30 min to a maximum rate of 400 mg/hr [20]. Due to IRRs, the protocol was amended. The first infusion was given over two days, 100 mg on day 1 and the remaining 900 mg on day 2 [20]. In addition, the rate of the infusion on day 1 was reduced to a constant rate of 25 mg/hr [20]. Therefore, the patients that experienced a reaction prior to the amendment likely contributed to the higher rate of reactions observed in the CLL11 clinical trial. The patients in our cohort received the first obinutuzumab infusion at an even slower rate compared to the CLL11 study. At CCMB, patients are started at an initial rate of 6 mg/hr on day 1 and this rate is doubled every hour to a max rate of 24 mg/hr [15]. The slower rate of infusion likely contributed to fewer severe reactions. In addition, there was a subset of 16 patients who received chlorambucil treatment prior to starting obinutuzumab as a means to decrease the lymphocyte count. Therefore, we suspect pre-treatment with CLB and slower infusion rates contributed to lower rates of severe infusion related reactions. This is in keeping with the study by Autore et al., which demonstrated a reduction in the severity of infusion related reactions with two cycles of chlorambucil pre-treatment. [21] In addition, the study by Lujan et al. showed that ibrutinib in combination with obinutuzumab can also reduce the severity of IRRs [22].

Most patients in our cohort were able to complete treatment with obinutuzumab. However, many patients were not able to tolerate the full dose of chlorambucil requiring dose reduction or discontinuation. Most often, this was due to myelotoxicity. In addition, a number of patients had chlorambucil doses held during treatment. Only 18 patients (26.9%) received all doses of obinutuzumab and chlorambucil. We may have expected this to have an impact on the PFS however this cohort had a similar median PFS (31.58 months) compared to the CLL11 clinical trial (31.3 months) [23]. Real world evidence published by Panovoska et al., demonstrated an event free survival of 49 months with chlorambucil and obinutuzumab. The rates of IRR were similar to the CLL11 study [24]. Rituximab and chlorambucil was used in our cohort in those either unwilling or unable to receive obinutuzumab. In this study, the PFS of patients treated with rituximab and chlorambuil was 20 months, however, the chlorambucil was given at 10 mg/m^2^ days 1–7 [24]. In another study that included over 430 patients treated with chlorambucil obinutuzumab in the frontline setting, the PFS was estimated at 27 months [25]. The median OS for our cohort has not been reached therefore it is unknown whether dose reducing the chlorambucil has impacted OS. The relative dose intensity (RDI) of obinutuzumab in the study by Herishanu et al. was 100% while for chlorambucil it was 74% [25]. In the CLL14 study the chlorambucil RDI was 94% suggesting patients are more likely to be dose reduced outside of clinical trials [26]. In our cohort, we were unable to calculate the RDI, but one explanation for dose reductions is that G-CSF is publically funded in Manitoba and only approved for primary prophylaxis in curative situations. Therefore, we may have seen dose reductions and interruptions more commonly due to rarer use of G-CSF. The fear of causing bone marrow failure in older individuals by pushing chemotherapy doses may also have led to more dose reductions. This could also explain the increased rates of infection in our cohorts, suggesting a role for primary prophylaxis with G-CSF in patients receiving obinutuzumab.

Older patients received less chlorambucil in our cohort, due to either toxicity, patient or physician preference. However, the older age group experienced similar rates of neutropenia and thrombocytopenia, lower rates of infection and higher rates of infusion related reactions and anemia compared to the younger age group. Therefore, the decision to hold chlorambucil may have been more influenced by patient or healthcare provider preference rather than adverse events. However, we are not able to answer this question through our retrospective review.

We cannot comment on whether adding chlorambucil to obinutuzumab improves survival in our cohort. Given the high level of toxicity, it might be more beneficial to give obinutuzumab alone. The literature on outcomes with single agent obinutuzumab is very limited. A randomized phase 2 study (GAGE) investigated the outcomes with 1000 mg and 2000 mg obinutuzumab monotherapy [27]. When compared to the combination of obinutuzumab and chlorambucil, this study found similar response rates and PFS in the 2000 mg obinutuzumab alone arm and lower response rates and shorter PFS in the 1000 mg obinutuzumab alone arm [27]. Therefore, it does not appear advisable to exclude chlorambucil for patients receiving the 1000 mg dose of obinutuzumab [27].

As mentioned previously, in Manitoba, obinutuzumab is infused at a slower rate compared to the CLL11 study. Therefore, it must be given over a longer duration which increases the costs, such as nursing time, associated with delivering the infusion. However, a slower rate of infusion likely lowers the severity of IRRs, which reduces costs associated with managing reactions. Since IRRs are uncommon after cycle 1 there has been recent literature investigating the use of rapid infusions starting with cycle 2. The GATHER and GATS studies have found a more rapid infusion rate (120 min and 90 min) to be safe in patients without grade 3 or higher IRRs with the standard infusion rate and a lymphocyte count less than 5 [28, 29]. We are currently evaluating the costs associated with this approach and adopting rapid infusions in CLL.

Recent studies are focusing on obinutuzumab in combination with ibrutinib or venetoclax, which are significantly more expensive than chlorambucil. Both the iLLUMINATE study and the CLL14 trial demonstrated significantly longer PFS in patients treated with ibrutinib-obinutuzumab and venetoclax-obinutuzumab respectively compared to chlorambucil-obinutuzumab [26, 30]. For the ibrutinib-obinutuzumab combination, the most notable benefit in PFS was in patients with high-risk disease (17pdel, TP53, del11q and IGHV unmutated) [30]. In addition, fewer patients treated with ibrutinib-obinutuzumab (4%) required additional treatment compared to those treated with obinutuzumab-chlorambucil (44%) with a median follow up of 31.1 months [30]. Patients also achieved greater depth of response with the ibrutinib-obinutuzumab and venetoclax-obinutuzumab combinations compared to obinutuzumab-chlorambucil [26, 30]. Therefore, despite the increased costs associated with these regimens, there may be selected patients, particularly those with high risk or bulky disease, where the benefit may justify the cost. Novel CLL directed therapies such as ibrutinib and acalabrutinib are used in the relapsed setting and as first-line therapy for high risk patients [31]. These are both oral medications taken daily and only discontinued if patients develop side effects or disease progression [4]. Many trials are assessing these oral agents in combination with obinutuzumab in the front-line and relapsed setting to improve depth of response in the marrow and ultimately, a time limited treatment with minimal toxicities [26, 30, 32].With so many novel agents and trials, substantial changes to treatment for CLL patients are likely to be implemented in the near future.

The limitations for this study include the retrospective study design and small cohort size. Due to the study design, there was variability in the treatment that patients received. The patients in this cohort did not all receive the same amount of chlorambucil which created difficulties with analyzing response and survival outcomes. Nevertheless, this heterogeneity allowed us to identify that pre-treatment with chlorambucil, through lowering lymphocyte counts, may help to reduce the severity of infusion related reactions. In addition, the cohort size for each of the treatment regimens and for the patients receiving pre-treatment with chlorambucil were small. Small group size impacts statistical power and may have contributed to a lack of statistical significance observed in our results. Unmeasured confounders are also possible due to the retrospective nature of the study.

## Conclusions

In contrast to many other centres, obinutuzumab-chlorambucil patients represented the largest group of front-line therapy in our cohort. Older patients with comorbidities can be treated with obinutuzumab-chlorambucil safely and with good outcomes in a non-trial environment. IRRs were less frequent and severe than previous published cohorts, likely related to a slower initial infusion rate and using chlorambucil to reduce the lymphocyte count prior to obinutuzumab. As a result, we have been able to treat the older frail patients who make up much of the CLL population. Reductions in chlorambucil dosage did not impact PFS or TTNT, however, longer follow up is needed to assess effects on OS. Neither age nor CIRS was a strong predictor of ability to tolerate therapy or progression free survival in our cohort.

## Supplementary Information

**Additional file 1: Table S1.** Treatment with obinutuzumab-chlorambucil based on age of 85 and CIRS score 10. **Figure S1.** Relationship between pre-treatment lymphocyte count and log odds of infusion-related reaction.

## Data Availability

PHIA legislation prevents the sharing of identifiable patient data. The small size in this study makes it difficult to adequately de-identify the data. Therefore, we cannot make the data available.

## Abbreviations

BR: Bendamustine-rituximab
CCMB: CancerCare Manitoba
CIRS: Cumulative illness rating scale
CLB: Chlorambucil
CLB/R: Chlorambucil-rituximab
CLL: Chronic lymphocytic leukemia
FCR: Fludarabine-cyclophosphamide-rituximab
FISH: Florescence in-situ hybridization
FR: Fludarabine-rituximab
IgHV: Immunoglobulin heavy chain variable region gene
IRR: Infusion related reaction
OB: Obinutuzumab
OS: Overall survival
PFS: Progression free survival
RDI: Relative dose intesnity
SD: Standard deviation
TTNT: Time to next treatment
