# Supplementary material for: Real world risk of infusion reactions and effectiveness of front-line obinutuzumab plus chlorambucil compared with other frontline treatments for chronic lymphocytic leukemia
Source: BMC Cancer. 2022 Feb 6;22:148. doi: 10.1186/s12885-022-09256-2 (PMC8818183; doi:10.1186/s12885-022-09256-2)
Supplement: Supplementary file 1 — Additional file 1: Table S1. Treatment with obinutuzumab-chlorambucil based on age of 85 and CIRS score 10. Figure S1. Relationship between pre-treatment lymphocyte count and log odds of infusion-related reaction. [file 12885_2022_9256_MOESM1_ESM.docx]

**Supplentary Table 1:** Treatment with obinutuzumab-chlorambucil based on ageof 85 and CIRS score 10

| **Treatment Characteristic** | | **Total** | | **Age at Treatment** | | | | **CIRS** | | |
| --- | --- | --- | --- | --- | --- | --- | --- | --- | --- | --- |
|  |  |  |  | **85+ (n=7)** | **Under 85 (n=60)** | | ***P*-value** | **10+ (n=17)** | **Under 10 (n=50)** | ***P*-value** |
| **Achieved Full Dose of Chlorambucil** | 26 (38.81) | | 1 (14.29) | | | 25 (41.67) | 0.23^a^ | 7 (41.18) | 19 (38.00) | 0.75^b^ |
| **Completed 6 Cycles of Obinutuzumab** | 46 (68.66) | | 4 (57.14) | | | 42 (70.00) | 0.67^a^ | 11 (64.71) | 35 (70.00) | 0.83^b^ |
| **Number of Chlorambucil Doses**  **Median (range)** | 10 (1-12) | | 4 (2-8) | | | 10.0 (1-12) | 0.09^c^ | 9 (2-12) | 10 (1-12) | 0.50^c^ |
| **Number of Obinutuzumab Doses**  **Median (range)** | 9 (1-9) | | 7 (2-9) | | | 9 (1-9) | 0.14^c^ | 9 (4-9) | 9 (1-9) | 0.89^c^ |

^a^ Fisher Exact test *P*-value

^b^ Chi-square *P*-value

^c^ Wilcoxon-Mann Whitney *P*-value

**Supplementary Figure 1:** Relationship between pre-treatment lymphocyte count and log odds of infusion-related reaction
